# Analysis of the mandibular molars root canals morphology. Study by computed tomography

**DOI:** 10.1590/0103-6440202205105

**Published:** 2022-10-21

**Authors:** Verônica de Oliveira Mantovani, Aline Evangelista de Souza Gabriel, Ricardo Gariba Silva, Ricardo Novak Savioli, Manoel Damião Sousa-Neto, Antonio M. Cruz-Filho

**Affiliations:** 1 Department of Endodontics, Ribeirão Preto Dental School, University of São Paulo, Ribeirão Preto, SP, Brazil

**Keywords:** Cone-beam computed tomography, mandibular first and second molars, root and canal anatomy

## Abstract

This study aimed to analyze the root and root canal morphology of mandibular first and second molars using CBCT images. A total of 2,400 mandibular molars exams were selected from 600 patients aged between 18 and 75 years. The number of roots, number of root canals, and root canal configuration according to the Vertucci classification were verified. Overall, 94.92% of mandibular first molars and 90.17% of mandibular second molars had two separate roots. Among the biradicular molars, the first molars showed a greater incidence of type IV canals in the mesial root and type I in the distal root. In the second molars, the most common canal form was type IV in the mesial root and type I in the distal root. In the triradicular molars, the type IV and type I configurations were the most common in the mesial root of the first molar and second molar, respectively. In both triradicular molars, there was a prevalence of type I canal in the distal and DL roots. Statistical analysis was performed at a significance level of 0.05. The number of roots was correlated with gender (Spearman test), and the canal's configuration with gender and bilaterality (Wilcoxon test). The subpopulation studied has a high incidence of bilateral symmetry and mandibular molars with two roots with two distinct mesial canals and one distal canal. The bilateral configuration is possible to estimate the number of canals, especially in images that are difficult to visualize, such as atresic canals.

## Introduction

Morphology variation of root canals has always been a challenge in the diagnosis and success of endodontic therapy (^1^). The endodontic intervention in all root canals is essential to promote cleaning, removal of necrotic content and reduction of the microbial contingent and their by-products (^2^,^3^). Thus, in-depth knowledge of the 3D internal morphology of the root canal is essential for those who practice Endodontics to define the treatment plan and execute the therapeutic protocol (^2^).

Different methodologies have been used to evaluate root canal morphology, such as scanning electron microscopy (^4^), and X-rays (^5^). However, these methods are destructive and present quality limitations since they provide two-dimensional images obtained from three-dimensional (3D) structures (^2^). The Cone beam computed tomography (CBCT) allows fast and accurate three-dimensional (3D) analysis of anatomical structures, in addition to being a non-invasive and non-destructive method (^6^,^7^). Clinically, it is a useful and precise tool in the anatomical details identification, enabling the ideal diagnosis and treatment (6). Considering its advantages, CBCT has become the standard method in several studies of root canal morphology (^8^,^9^,^10^). These studies have shown that the number and classification of root canals can vary in each group of teeth, according to with ethnicity, sex and age (^8^,^9^,^10^,^11^). Lower first molars usually have two roots with two distinct canals in the mesial root and a single distal canal. Reports in the Indian population showed that the number of canals varied from 1 to 6 in the lower first molars (^12^,^13^). The presence of an extra-distolingual root (also known as radix entomolaris) has been described in Mongolians (^14^). In the Korean population, the prevalence of this root occurs most often in the first molars and regularly bilaterally (^15^).

From the genetic and ethnic point of view, the current Brazilian population is very diverse and is considered one of the most heterogeneous populations in the world. Its constitution received important genetic contributions from four main continental groups: Europeans, Africans, Asians, and Native Americans (^6^). Most of the studies found in the literature are directed to populations of Caucasoid origin, not being applicable to heterogeneous populations such as the Brazilian. Few studies have comprehensively evaluated the root canal morphology of first and second molars simultaneously in ethnic populations using CBCT imaging (^6^).

Although previous studies (^6^,^16^) have already investigated the internal morphology of lower molars in the Brazilian population, the sampling of these studies was very small, which can make the result inconsistent. The present study was designed to analyze the canal morphology and the incidence of bilaterality, number of roots and canals of permanent mandibular first and second molars in a Brazilian subpopulation by retrospectively analyzing a significant number of "in vivo" CBCT images.

## Materials and methods

### Sample selection

The local research ethics committee approved this study (CAAE #42498821.8.0000.5419). CBCT images of mandibular first and second molars were collected from patients who underwent computed tomography (CT) at a School of Dentistry. CBCT examination was indicated when the information generated by conventional X-ray images was insufficient for a conclusive diagnosis. No image was generated exclusively for this study. A total of 6,360 CT scans were analyzed and 2,400 scans were selected. Selection criteria included scans of patients who had bilateral permanent mandibular first and second molars with fully developed roots, without resorption, and with good image quality in the area of interest. Images with previous endodontic treatment, intraradicular retainers and/or metal crowns, teeth with incomplete rhizogenesis or apical periodontitis and any other factors that hindered the detailed visualization of the image were excluded from the study. The sample comprised 600 patients, 215 male (35.83%) and 385 female (64.17%), aged between 18 and 75 years (mean age, 27.6 years).

**Figure 1:**
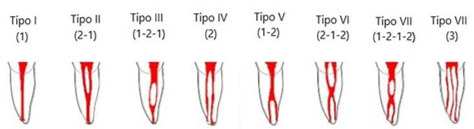
Classification of root canal configuration according to Vertucci.

Imaging analysis was performed using the Orthopantomograph OP300 device (Instrumentarium Dental, Tuusula, Finland) with 360° head rotation. All examinations were conducted according to the manufacturer's standards, with constant exposure parameters: tube voltage of 85 KVp, current of 8 mA and focal spot of 0.5×0.5 mm, and total filter of 3.52 mm Al. The exposure used was the minimum necessary for correct image quality. The data stored in DICOM format (digital imaging communication in medicine) were analyzed with the aid of *RadiAnt DICOM* Viewer 2020 5.5.1 on a 14-inch monitor (Inspiron 14R 54205.1, Dell Computadores do Brasil, Brazil), with a resolution of 1366×768 pixels, in a dark environment. Roots and root canals were observed in sagittal and axial sections, with planes 30-mm wide and 0.5-mm thick. Axial navigation followed in 0.1-mm×0.1-mm sections from coronal to apical as well as from apical to coronal. In teeth with more than two roots, the axial navigation was customized for each root. The quali-quantitative evaluation was performed by two examiners calibrated using the Kappa test (0.872), an endodontist and a radiologist, with experience in manipulation and image diagnosis, independently and in two sessions (15 days apart, dark, free from audible noise). In disagreement on the interpretation of the images, the two examiners reached a common agreement. The number of roots, the number of canals and canal configurations based on the Vertucci classification (^11^) (Fig. 1), and the bilateral occurrence rate of anatomical variations were identified, considering gender. conforme o gênero. The parameters evaluated for each patient were recorded in a duly identified individual spreadsheet. Subsequently, a file was set up with the data referring to each three-dimensional parameter for statistical processing.

Statistical analysis was performed using SPSS (Version 28.0.0; SPSS Inc, IBM, USA), with significance set at p < 0.05. Spearman's correlation test was applied to verify the correlation between sex and the number of roots. Wilcoxon test compared canal configuration with sex and bilateral configuration.

## Results

### Number of roots on the mandibular first and second molars

The number of roots according to side and sex is listed in Table 1. Only 12 (1.0%) mandibular first molars (MFMs) had one root, all in female patients. The incidence of mandibular second molars (MSMs) with one root was 4.88% (21-430) in male patients and 9.74% (75-770) in female patients.

The incidence of MFMs with two roots was 96% (576-600) and 93.83% (563-600) on the right and left side, respectively, and 95.81% (412-430) and 94.41% (727-770) in male and female patients, respectively. In MSMs, the incidence of two roots was 90% (540-600) and 90.33% (542-600) on the right and left side, respectively, and 94.42% (406-430) and 87.79% (676-770) in male and female patients, respectively (Table 1). No difference was found between sexes in the frequency of two roots in the mandibular first (*p* = 0.608) and second molars (*p* = 0.614).

Forty-nine (4.08%) MFMs had three roots: 4.19% (18-430) in male patients, and 4.02% (31-770) in female patients. Only 22 (1.83%) MSMs had three roots.

**Table 1:** The frequency distribution and percentage of the number of roots in mandibular first and second molars based on laterality and patient gender.

Number of roots		Left			Right			
		Female	Male	Number of teeth	Female	Male	Number of teeth	Total number of teeth
First Molar	1 root							
	Conical	-	-	-	3	-	3 (0,50)	3 (0,25)
	C-shaped	6	-	6 (1,00)	3	-	3 (0,50)	9 (0,75)
	Total			6 (1,00)			6 (1,00)	12 (1,00)
	2 roots							
	2 canals	3	-	3 (0,50)	7	-	7 (1,17)	10 (0,83)
	3 canals	357	203	560 (93,33)	360	209	569 (94,83)	1129 (94,08)
	Total			563 (93,83)			576 (96,00)	1139 (94,92)
	3 roots							
	3 canals Total	19	12	31 (5,17) 31 (5,17)	12	6	18 (3,00) 18 (3,00)	49 (4,08) 49 (4,08)
Second Molar	1 root							
	1 canal	15	3	18 (3,00)	16	3	19 (3,16)	37 (3,08)
	C-shaped	19	9	28 (4,66)	25	6	31 (5,16)	59 (4,91)
	Total			46 (7,66)			50 (8,34)	96 (8,00)
	2 roots							
	2 canals	48	13	61 (10,16)	33	16	49 (8,16)	110 (9,16)
	3 canals	294	187	481 (80,18)	301	190	491 (81,86)	972 (81,00)
	Total			542 (90,34)			540 (90,00)	1082 (90,17)
	3 roots							
	3 canals Total	9	3	12 (2,00) 12 (2,00)	10	-	10 (1,66) 10 (1,66)	22 (1,83) 22 (1,83)

### Mandibular first molar root canal configuration

The results of the root canal configurations are presented in Tables 2 and 3. The incidences of different root canal configurations did not differ between females and males (*p* = 0.941).

Besides the root canal configuration based on Vertucci's classification (^11^) (Figure 1), we also observed that 9 (0.75%) uniradicular MFMs presented C-shaped canal (Table 1) and 3 (0.25%) teeth had type I configuration (Figure 2A).

**Figure:**
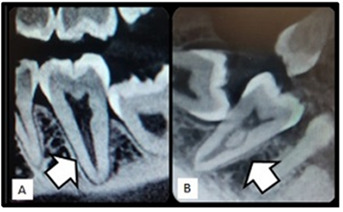

There was a wide variety of canal configurations in the mesial and distal roots of MFMs with two roots. We observed a higher incidence of type IV configuration in the mesial root (785-1139, 68.92%), and type I in the distal root (947-1139, 83.14%) (Table 2). This configuration showed that in general, the MFM presents two canals in the mesial root and one canal distally. In mandibular molars with three roots, the root arrangement showed one mesial root and two distal roots (distal, D; distolingual, DL). Different canal configurations were seen in the mesial root; type IV was the most common (27-49, 55.10%). In the D and DL roots, there was a 100% prevalence of type I configuration (49-49, 100%) (Table 3).

### Mandibular second molar root canal configuration

Fifty-nine (4.91%) uniradicular MSMs of both sexes had a C-shaped canal (Table 1). Among them, 25 (2.08%), 6 (0.5%), 3 (0.25%), and 3 (0.25%) teeth had type I, type III (Figure 2B), type II, and type VII configurations, respectively.

Most of the 1,082 MSMs with two roots had a type IV canal configuration (41.40%) in the mesial root. In the distal root, 99.16% (1,073-1,082) showed a type I canal (Table 2). According to the configuration observed, the MSMs with two roots tends to present two different mesial canals and one distal canal. MSMs with three roots presented 100% (22-22) of cases of type I configuration in the three root canals (Table 3).

### Bilateral symmetry occurrence

Bilateral symmetry was considered when the shape of the canal of a given molar, whether with one, two or three roots, was similar to the homologous tooth. Bilateral symmetry was found in 88.83% (533-600) of MFMs, (*P =* 0.003) and in 53.16% (319-600) of MSMs (*P =* 0.02).

**Table 2:** The frequency and percentage of configuration of root canal system in 2-rooted mandibular first and second molars based on location and gender.

		Type I (1-1)	Type II (2-1)	Type III (1-2-1)	Type IV (2-2)	Type V (1-2)	Type VI (2-1-2)	Total
First Molar	Mesial root	10 (0,89)	284 (24,93)	26 (2,28)	785 (68,92)	34 (2,98)	-	1139
	Female	10	182	19	500	16	-	727
	Male	-	102	7	285	18	-	412
	Distal root	947 (83,14)	93 (8,16)	33 (2,90)	33 (2,90)	33 (2,90)	-	1139
	Female	605	51	24	24	23	-	727
	Male	342	42	9	9	10	-	412
Second Molar	Mesial root	110 (10,16)	360 (33,27)	113 (10,44)	448 (41,40)	48 (4,45)	3 (0,27)	1082
	Female	81	229	70	262	31	3	676
	Male	29	131	43	186	17	-	406
	Distal root	1073 (99,16)	3 (0,28)	3 (0,28)	-	3 (0,28)	-	1082
	Female	667	3	3	-	3	-	676
	Male	406	-	-	-	-	-	406

**Table 3:** The frequency and percentage of configuration of root canal system in 3-rooted mandibular first and second molars based on location and gender.

		Type I (1-1)	Type II (2-1)	Type III (1-2-1)	Type IV (2-2)	Total
First Molar	Mesial root	9 (18,38)	7 (14,28)	6 (12,24)	27 (55,10)	49
	Female	3	4	6	18	31
	Male	6	3	-	9	18
	DB root	49 (100,00)	-	-	-	49
	Female	31	-	-	-	31
	Male	18	-	-	-	18
	DL root	49 (100,00)	-	-	-	49
	Female	31	-	-	-	31
	Male	18	-	-	-	18
Second Molar	Mesial root	22 (100,00)	-	-	-	22
	Female	19	-	-	-	19
	Male	3	-	-	-	3
	DB root	22 (100,00)	-	-	-	22
	Female	19	-	-	-	19
	Male	3	-	-	-	3
	DL root	22 (100,00)	-	-	-	22
	Female	19	-	-	-	19
	Male	3	-	-	-	3

## Discussion

This study evaluated the number of roots and root canal configuration according to Vertucci's classification (^11^) and the influence of sex on canal configuration of 2,400 mandibular molars in a Brazilian southeastern subpopulation. We also verified whether the anatomical variation of the sample occurs bilaterally. This study provides more complete information, and superior sample size when compared to previous studies of mandibular molars in the Brazilian subpopulation (^6^,^16^). Wider sampling than previous studies (^6^,^16^) may provide more reliable information on root canal morphology (^8^). The analysis was based on CBCT images of 600 patients of both sexes, aged 18-75 years. A total of 600 images were obtained from each mandibular molar of patients from the dental college in need of different diagnostic purposes. CBCT was requested only when strictly necessary aiming at the lowest risk/benefit to the patient. “In vivo” CBCT was a very useful tool in the investigation of root canal configuration.

MFMs are usually described as having two roots, namely mesial and distal, and three root canals (^6^,^8^,^16^). This configuration has been observed in different populations such as Spanish (94% with two roots and three canals) (^9^), and Thai (74.7% with two roots and 56.1% with three canals) (^14^). In the present investigation, we found 1,139 (94.92%) first molars with two roots: 727 (63.83%) in female and 412 (36.17%) in male participants. The analysis of the internal anatomical configuration of these teeth showed that 1,103 (91.91%) had three canals. Our findings show that the number of roots and canals for the MFMs, both right and left follow a similar description to those reported for different populations (6,9,14,16). The number of patients with two roots (1,082, 90.16%) and three root canals (1,116, 93%) in MSMs was similar to the findings for MFMs. However, a study conducted in an Indian subpopulation reported a high prevalence of the second molar with two roots (288-345, 83.4%) and only two root canals (248-345, 71.8%) (^17^). The method performed in the study used 345 extracted teeth and the diaphanization technique. Diaphanization is an invasive and limited method that can lead to misinterpretation. More recent 3D analyses using CBCT for evaluation of root morphology reported prevalence of second molars with two roots and three canals in populations of Indian (79.35%) (^18^), and Brazilian (54%) (^6^).

In this study, we found a rather low incidence of mandibular molars with a single root, with 87 (3.62%) in female and 21 (0.87%) in male participants, as well as with three roots, 50 (2.08%) in female and 21 (0.87%) in male participants. The low incidence of molars with one or three roots in different populations was reported by previous studies (^6^,^9^,^19^). Although a great part of the population presents the pattern of two roots and three root canals to describe the mandibular molars, such findings cannot be generalized. Several studies have shown a prevalence of up to 40% of mandibular molars with three roots in Asians (^8^,^14^,^19^) and of four roots in other populations (^20^). A higher incidence of molars with three roots is observed in Mongolians than in people of white and black origin (^21^). Thus, dental surgeons should be aware of the possibility of an extra root, especially during the care of patients of Asian origin.

There was divergence in the results of the influence of sex on the number of roots. There are reports of a higher prevalence of first molars with two roots in Chinese male participants (77.1%) than in female participants (69.3%) (^19^). Although we found first molars with a single root only in female participants (6, 1%), overall, there was no correlation between the number of roots and sex (ρ = 0.019). Our findings seem to be consistent because recent studies with CBCT and with large sampling performed in Turkish (^20^), and Chinese (^19^) subpopulations showed similar results. In our study, more than 70% of first molars and more than 65% of second molars showed bilaterality regarding the number of roots. The high incidence suggests that the clinician should consider the possibility of bilateral roots especially when treating the Brazilian population.

According to the literature, the internal configuration of root canals of mandibular molars varies according to the population and methodology used (^11^,^19^). Study reported that practically half of the sample of mesial roots present a type IV configuration (^22^). In the present study, the prevalence of type IV canals in MFMs with two roots was 68.77% (500-727) in female and 69.17% (285-412) in male participants, similar to previous studies (8,16,23). Wang et. al. (^19^), in a study conducted in Western Chinese individuals, mentioned an even higher incidence (94%). However, a study in Spain showed the prevalence of type II configuration (78.2%) in the mesial root. The divergence of the results may be associated with the study design (in vivo vs in vitro), ethnic difference, and the visualization technique of the canals (CBCT and bleaching) (^19^). Our study revealed that MSMs with two roots showed type IV configuration in the mesial root, with an incidence slightly lower than that found in MFMs, with 676 (53.25%) in female and 225 (55.41%) in male participants. A previous study agrees with our result (^23^). In the distal root, the canal configuration was type I both in MFMs (606 [83.2%] in female and 342 [83%] in male participants) and MSMs (667 [98.66%] in female and 406 [100%] in male participants). According to the literature, type I configuration in the distal root of mandibular molars is predominant (^8^,^9^,^19^,^23^). The presence of three canals was around 10% of the sample and we did not identify distal roots with three canals. Although the incidence is low, the presence of three canals in the distal root seems to be characteristic of African populations (3.0%) (^24^). In our study, the incidence of bilateral canal configuration was high. Such data can help the professional in the identification of the number of canals and foramens between homologous teeth.

In this study, among the total number of single-rooted mandibular molars (108-2,400, 4.5%), 62.96% (68-108) presented a "C" canal. This configuration seems to be prevalent in mandibular molars with one root (^8^,^18^,^23^). Pawar et. al. (^18^) found that 100% of uniradicular molars of Indians had "C" canals. In mesial roots of MFMs with three roots, there was a prevalence of type IV configuration (27-49, 55.10%), which agrees with other studies (^8^,^16^). On the other hand, we found the prevalence of type I configuration (32-32, 100%) in the mesial of MSMs with three roots. There are few studies on the internal morphology of MSMs that point out a higher incidence of type IV configuration in the mesial root (^6^). The divergence of results may be related to ethnic differences or sample size. All distal and distolingual roots of mandibular molars presented a type I configuration (100%). These findings seem to be consistent with studies performed with CBCT in different populations (^6^,^8^,^16^).

The CBCT images analyzed in this study showed lower molars with two distinct roots and three root canals, with a high incidence of bilaterality. The observation of bilaterality becomes essential in treating tooth homologs with restrictions on radiographic examinations or difficulty interpreting radiography. CBCT quality is high enough to visualize the root canal morphology for endodontic treatment. However, dental professionals should not use it routinely. CBCT is an expensive test, and the patient's exposure to radiation must justify the benefits achieved and overcome the potential risks (^6^,^9^).

## Conclusions

This retrospective in vivo CBCT study found that in a Brazilian subpopulation from the southeast of the country, mandibular molars usually have two roots with two different mesial canals and one distal canal. We also found a high incidence of bilateral symmetry, especially in the MFM, which can be a reference for determining the internal anatomy based on the canal configuration of homologous teeth.
